# How Does Employee Dependence on Intelligent Machines Influence Turnover Intention? A Serial Mediation via Workplace Objectification and Workplace Loneliness

**DOI:** 10.3390/bs16091475

**Published:** 2026-08-25

**Authors:** Xianwei Zheng

**Affiliations:** School of Management, College of Science and Technology, Ningbo University, Ningbo 315300, China; zhengxianwei@nbu.edu.cn

**Keywords:** dependence on intelligent machines, workplace objectification, workplace loneliness, turnover intention, conservation of resources theory

## Abstract

While integrating intelligent machines enhances workplace efficiency, it paradoxically correlates with increased employee turnover. To explain this efficiency–turnover paradox, this study draws primarily on conservation of resources theory to investigate how employees’ dependence on intelligent machines triggers turnover intention. Using data from a two-wave longitudinal survey with a two-month lag among 241 manufacturing employees in China, we tested a serial mediation model. Results indicate that dependence on intelligent machines does not directly predict turnover intention. Instead, it exerts an indirect effect through a resource-depleting serial mechanism. By transferring human–machine interaction patterns to interpersonal relations, machine dependence triggers workplace objectification, which in turn exacerbates workplace loneliness. Faced with this severe relational and emotional depletion, employees develop turnover intention as a self-protective strategy. By integrating technological, socio-cognitive, emotional and behavioral dimensions, the findings demonstrate that digital reliance undermines employee retention primarily through a cascade of interpersonal objectification and emotional loneliness, underscoring the necessity of techno-humanistic management practices.

## 1. Introduction

Digital transformation has repositioned intelligent machines from auxiliary tools to functioning as embedded “digital colleagues” in everyday work processes (Bankins et al., 2024; Danatzis et al., 2025). This human–machine integration restructures how employees carry out their tasks and collaborate with others. As dependence on intelligent machines intensifies, employees’ dependence on human coworkers correspondingly declines (Tang et al., 2023a). This development gives rise to a paradox: while deep technological integration improves efficiency, it simultaneously generates psychological costs. Employees report heightened job insecurity, workplace loneliness, and burnout resulting from dependence on intelligent machines (He et al., 2023; Tang et al., 2023a; Yam et al., 2023). Whether these psychological costs translate into turnover intention and ultimately destabilize employment relations remains underexplored. Practitioners have raised concerns about this potential progression, but empirical research on the link between dependence on intelligent machines and turnover intention remains scarce.

Dependence on intelligent machines introduces new risks that reach beyond conventional adaptation challenges. At its core, this phenomenon erodes employees’ psychological resources (He et al., 2023; Meng et al., 2025). Conservation of resources theory (COR theory; Hobfoll, 1989) suggests that when recovery appears unlikely, perceived depletion of critical psychological resources elicits self-protective withdrawal responses. Turnover intention represents a typical withdrawal response, often seen in employees experiencing prolonged resource loss (Griffeth et al., 2000; A. Wu et al., 2026). Yet empirical examinations of this mechanism remain limited. The specific mechanisms through which dependence on intelligent machines heightens turnover intention have yet to be adequately examined.

Despite growing scholarly attention, research on dependence on intelligent machines has documented negative effects in multiple domains, yet explanatory frameworks remain fragmented and largely unintegrated. Existing studies tend to examine human–machine dynamics, peer interactions, individual psychological states, and organizational outcomes as distinct domains of inquiry. At different analytical levels, prior studies have emphasized separate focal points: research on human–machine dynamics has addressed technological trust deficits (Mirowska & Arsenyan, 2025); peer-level investigations have examined the erosion of interpersonal interactions (Yam et al., 2023); individual-level studies have centered on mental-health impairment (Tang et al., 2023a); and organizational analyses have explored reconfigured mechanisms of control and collaboration (Leonardi & Treem, 2020). This compartmentalized approach overlooks a crucial dynamic. Pressures created by dependence on intelligent machines tend to spill over from human–machine interactions into coworker relationships, gradually depleting employees’ psychological resources. The lack of integrated causal models creates an unresolved empirical puzzle: productivity gains in human–computer collaborative settings appear to coincide with elevated turnover intention among employees. To explain this paradox, researchers need to move beyond single-level analyses and adopt frameworks that capture the full process from technological factors to socio-cognitive, emotional, and behavioral outcomes.

To address this gap, this study draws primarily on Conservation of Resources (COR) theory (Hobfoll, 1989) to develop a serial mediation model. We position COR theory as the overarching framework to explore the fundamental motivational drivers of turnover intention. A central tenet guiding our conceptualization of the direct relationship is COR’s Primacy of Loss principle, which postulates that resource loss generally carries more psychological weight than resource gain (Hobfoll et al., 2018). Drawing on this principle, we theorize that the profound loss of core agency caused by dependence on intelligent machines is likely to eclipse potential gains in operational autonomy, creating a net resource depletion that might compel employees to exit the organization. Beyond this direct resource-drain process, our model further explicates a sequential interpersonal mechanism. As employees increasingly treat coworkers as mere instruments in highly automated work environments, this active workplace objectification is expected to destroy reciprocal connections and precipitate profound workplace loneliness. Ultimately, by mapping these direct and indirect pathways within a COR-based framework, we explore whether the continuous depletion of critical psychological and social resources prompts employees to utilize turnover intention as a defensive coping strategy.

Building on this theoretical foundation, the study introduces four interrelated constructs within an integrative analytical framework: dependence on intelligent machines (technological), workplace objectification (socio-cognitive,), workplace loneliness (emotional), and turnover intention (behavioral). The framework delineates a complete process linking technological exposure with employee withdrawal behavior. By specifying the micro-level psychological mechanisms through which dependence on intelligent machines affects turnover intention, this research provides a theoretical perspective to explain employee responses to digital transformation.

## 2. Literature Review and Hypotheses

### 2.1. Dependence on Intelligent Machines and Turnover Intention

Dependence on intelligent machines refers to a situation in which employees may, as a function of the design of their job, at times need to rely on the sophisticated work capability of intelligent machines to augment their ability to make decisions, execute tasks, and monitor ongoing work (Tang et al., 2023b). Therefore, it essentially represents a structural, task-based reliance rooted in job design, rather than a subjective psychological state. Research on performance outcomes has demonstrated two contrasting effects. Dependence enhances task performance when employees perceive accelerated goal progress or heightened role-breadth self-efficacy, but dependence diminishes performance when employees experience threats to self-esteem or heightened role ambiguity (Tang et al., 2022, 2023b). The mechanisms linking dependence on intelligent machines to employees’ broader attitudes and behaviors remain underexplored. In particular, little is known about how this dependence relates to turnover intention.

To elucidate the mechanisms linking this structural job characteristic to turnover intention, we bridge the theoretical gap between objective job design and subjective psychological states using the multidimensional conceptualization of work autonomy (Deci & Ryan, 1985; Morgeson & Humphrey, 2006). We posit that dependence on intelligent machines paradoxically alters employees’ subjective autonomy. On one hand, by reducing reliance on human coworkers (Hai et al., 2025; Yang, 2025), the continuous availability of intelligent systems affords greater task flexibility (Schinoff et al., 2020; Shin et al., 2025), thereby enhancing employees’ perceived work scheduling and method autonomy (Breaugh, 1985). Conversely, the algorithmic substitution of human cognitive judgment (S. Chen et al., 2025; Sparascio et al., 2023) compels employees to surrender critical task control, severely depriving them of decision-making autonomy (Morgeson & Humphrey, 2006). Consequently, this structural dependence generates opposing psychological effects: enhanced scheduling and method autonomy (potential resource gain) versus diminished decision-making autonomy (resource loss).

To resolve this tension, we draw upon COR theory to argue that not all forms of autonomy hold equal value as psychological resources. Machine-enhanced operational autonomy (e.g., scheduling or method autonomy) serves merely as a peripheral resource. Its utility is fundamentally compromised because substituting human collaboration with AI induces mechanistic dehumanization and deprives employees of essential social support (Bender, 2024; Li et al., 2026). Conversely, decision-making autonomy is a central, high-value resource integral to professional identity and agency; surrendering core cognitive tasks to algorithms profoundly threatens it (Turkkan Zencirli & Altin, 2025). Crucially, COR’s Primacy of Loss principle dictates that resource loss carries substantially more psychological weight than resource gain (Hobfoll et al., 2018). Consequently, the severe depletion of core decision-making agency eclipses any superficial gains in operational autonomy, resulting in a net depletion of psychological resources. To halt this downward spiral, employees may resort to defensive coping strategies, primarily utilizing turnover intention as a form of psychological withdrawal to preserve remaining resources (Hom et al., 2012; Hua & Bao, 2025). Thus, we propose the following:

**H1.**  *Employee dependence on intelligent machines positively predicts turnover intention*.

### 2.2. The Mediating Effect of Workplace Objectification

Workplace objectification involves treating others as mere instruments while denying their fundamental human qualities (Baldissarri et al., 2022). Rather than relying on multiple disparate theoretical perspectives, we argue that dependence on intelligent machines inherently fosters workplace objectification by directly triggering its two core dimensions: instrumentality and dehumanization (Baldissarri et al., 2017, 2022). First, regarding instrumentality, structural dependence on intelligent machines forces employees into an environment that prioritizes relentless efficiency, data-driven task completion, and heightened performance standards (Tang et al., 2023b). Through repeated interactions with these task-driven systems, employees develop a learned cognitive pattern focused entirely on utility (Zhang et al., 2026). As this efficiency-oriented schema spills over into everyday work, employees begin to evaluate their human peers solely based on their functional value for task completion (Belmi & Schroeder, 2021; S. Chen et al., 2025). In highly competitive and homogenized automated environments, colleagues are increasingly reduced to mere tools or functional assets used to safeguard one’s own productivity (S. Chen et al., 2025).

Second, regarding dehumanization, dependence on intelligent machines fundamentally alters the benchmark for interpersonal evaluation. As the infallible and emotionless execution of automated systems becomes the daily standard, employees implicitly begin comparing their human coworkers against these artificial agents (W. Wu & Wang, 2025). Against this artificial baseline, natural human characteristics such as emotional needs, psychological fluctuations, and normal performance variations are increasingly perceived as inefficiencies or operational flaws (Turkkan Zencirli & Altin, 2025). This comparative process triggers assimilation-induced dehumanization (Kim & McGill, 2023), driving employees to actively devalue the warmth, individuality, and distinctive human qualities of their peers. Consequently, as interactions with intelligent systems increasingly dictate work routines, individuals internalize this mechanistic logic and seamlessly translate human–AI interaction patterns into interpersonal relationships (Dang & Liu, 2025). By denying the fundamental humanness of their colleagues, reliance on such technologies directly increases employee workplace objectification (Yang, 2025). Taken together, through the dual pathways of instrumentality and dehumanization, dependence on intelligent machines firmly establishes workplace objectification.

Consequently, workplace objectification acts as a critical catalyst for employee turnover intention. Drawing on Conservation of Resources (COR) theory, viewing colleagues merely as tools neglects human emotional bonds, which severely depletes employees’ psychosocial resources and diminishes their perceived authenticity (Cheng et al., 2022). Experiencing this profound relational impoverishment creates an alienating work climate, ultimately motivating employees to exit the organization to protect themselves from further psychological depletion (Hobfoll et al., 2018). Furthermore, workplace objectification replaces high-quality interpersonal interactions with cold, transactional exchanges. Treating peers strictly as functional assets fosters unfavorable social behaviors, such as social undermining and workplace incivility (Belmi & Schroeder, 2021; Zhang et al., 2026). This shift inevitably triggers toxic cycles of mistreatment, prompting employees to physically and psychologically withdraw from the deteriorating social environment (Baldissarri et al., 2022). Ultimately, the instrumental rationality intensified by dependence on intelligent machines destroys the relational foundation of the workplace, thereby elevating turnover intention. Therefore, we propose the following:

**H2.**  *Workplace objectification mediates the relationship between dependence on intelligent machines and turnover intention*.

### 2.3. The Mediating Effect of Workplace Loneliness

The Process Model of Workplace Loneliness posits that workplace loneliness emerges when actual social relationships fall significantly short of desired relationships (S. Wright & Silard, 2021). Dependence on intelligent machines increasingly alters daily work experiences in ways that further widen this gap. Intelligent machines lack emotional intelligence and often act as substitutes for human collaborators, reducing interpersonal emotional communication and limiting avenues for obtaining emotional resources (Lv et al., 2024; Meng et al., 2025). These changes thereby weaken actual social relationships, generating workplace loneliness. Empirical evidence supports this process: increased interactions with artificial intelligence are found to heighten social disconnection and intensify workplace loneliness (Tang et al., 2023a).

Workplace loneliness has been shown to generate negative consequences for employees (Bryan et al., 2023), including heightened turnover intention (Gilmer et al., 2023; Sasaki et al., 2025). A self-reinforcing cycle underlies these maladaptive outcomes. Workplace loneliness is aversive, impairs well-being, and prompts self-isolation behaviors that further deepen social isolation (Gabriel et al., 2021; Tang et al., 2023a). COR theory explains these withdrawal responses. Resource depletion resulting from workplace loneliness activates defensive mechanisms, thereby prompting employees to consider leaving due to diminished social connection and a sense of work meaninglessness (Ghadi, 2017; Hobfoll et al., 2018; Lam & Lau, 2012).

Dependence on intelligent machines reduces workplace social relationships by limiting emotional communication and access to emotional resources, which in turn fosters workplace loneliness. Workplace loneliness triggers a self-reinforcing cycle in which resource depletion evokes defensive coping responses, including turnover intention. Therefore, we hypothesize that

**H3.**  *Workplace loneliness mediates the relationship between dependence on intelligent machines and turnover intention*.

### 2.4. The Serial Mediating Effects of Workplace Objectification and Workplace Loneliness

Dependence on intelligent machines may influence turnover intention via workplace objectification (H2) and workplace loneliness (H3). These mechanisms may coexist, as theoretical and empirical evidence suggests that workplace objectification can trigger workplace loneliness. Indeed, we conceptualize this specific objectification–loneliness link as the central theoretical pivot of our research model. Accordingly, a serially mediated process may operate. Dependence on intelligent machines first increases workplace objectification. This increase, in turn, fosters workplace loneliness and ultimately elevates turnover intention.

Widespread intelligent machine adoption fosters work environments characterized by algorithmic control, where organizations use recording, rating, restricting, and recommending systems to monitor and evaluate work processes (Kellogg et al., 2020). As a result, this tool-efficiency orientation shifts employees’ focus from social interaction to performance metrics. Because healthy workplace interactions rely heavily on reciprocal exchange and mutual trust (Cropanzano & Mitchell, 2005), objectifying behaviors severely undermine this reciprocal foundation. Instrumental treatment of colleagues signals relational detachment and emotional indifference. Such perceptions often trigger defensive responses, which include fewer helping behaviors and discourteous treatment (Baldissarri et al., 2022; Yang, 2025). Consequently, the quality of social interaction declines, and interpersonal bonds weaken systematically. Furthermore, because employees possess a fundamental human need to form stable, positive emotional connections (Baumeister & Leary, 1995), when actual workplace relationships fall significantly short of these expectations due to mutual objectification, this unmet need for belonging generates workplace loneliness (S. Wright & Silard, 2021). Empirical evidence confirms that weaker workplace social connections intensify workplace loneliness (Basar et al., 2024; Kuriakose et al., 2023), whereas stronger connections relieve it (Jin & Ikeda, 2024; Labella & van Knippenberg, 2024).

Taken together, the direct relationship between workplace objectification and workplace loneliness bridges the cognitive and emotional phases of employee experiences. Dependence on intelligent machines may sequentially foster workplace objectification and loneliness, thereby increasing turnover intention through a serial mediation sequence. In summary, we hypothesize that

**H4.**  *Workplace objectification and workplace loneliness sequentially mediate the relationship between dependence on intelligent machines and turnover intention*.

**Figure 1:**
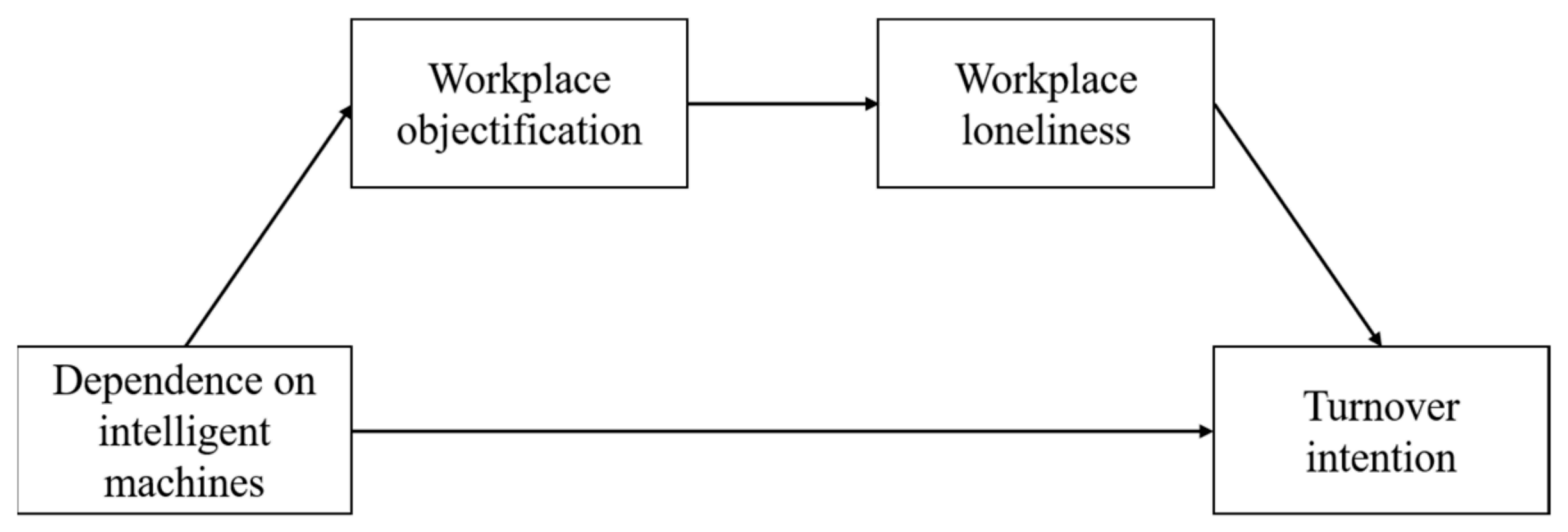
Research model.

All proposed hypotheses are depicted in Figure 1.

## 3. Research Methods

### 3.1. Research Context, Sample and Procedures

To ensure the external validity of our findings, data were collected from five manufacturing enterprises in China that have actively implemented digital transformation and smart manufacturing initiatives. These enterprises operate in advanced manufacturing sectors, producing textile machinery, electrical appliances, and mechanical accessories. The manufacturing sites are characterized by a high overall integration of intelligent machinery, including automated assembly lines, collaborative robots (cobots), and computer numerical control (CNC) systems.

Participants in this study were primarily frontline employees across various production workshops. As end-users of these technologies, they execute tasks dictated by or alongside machines, possessing limited autonomy to manage or reconfigure the underlying AI systems. Crucially, selecting these enterprises provided an ideal setting to test our theoretical model because the level of dependence on intelligent machines varied significantly across different workshop roles. The sample captured a complete spectrum of human–machine collaboration: at one extreme were roles relying entirely on traditional manual labor without machine interaction; at the other extreme were roles fully automated where employees acted solely as system monitors; and in between were various roles requiring constant physical and cognitive coordination with intelligent machines. In these digitized workshops, employees with a high dependence on intelligent machines are often required to remain fixed at monitoring stations or interact continuously with screens and cobots. This setup physically and attentionally separates them from human peers, drastically disrupting traditional shop-floor socialization. Consequently, this specific manufacturing environment makes it highly salient to observe how technological reliance alters interpersonal behaviors and triggers psychological isolation, providing a perfectly matched context to test our entire serial mediation model.

Researchers coordinated with human resources directors to obtain employee rosters and helped administer on-site questionnaire surveys during break times. Participants received an explanation of the survey’s purpose, information about voluntary participation, and assurance of anonymity. Questionnaires were distributed and returned in sealed envelopes to ensure confidentiality. To minimize common method bias, a two-stage longitudinal design with a two-month interval was used. Stage 1 focused on measuring dependence on intelligent machines, workplace objectification, and demographic information. Stage 2 then examined workplace loneliness and turnover intention.

A total of 400 questionnaires were distributed in Phase 1, yielding 327 returns (81.8% response rate). Two months later, follow-up questionnaires were sent to the Phase 1 respondents, obtaining 241 matched valid responses (73.7% retention rate). The demographic characteristics of the sample were as follows. For gender, 66.0% were male, and 34.0% were female. For age, 49.0% were under 30, 37.3% were between 30 and 40, 11.2% were between 41 and 50, and 2.5% were over 50. For education, 65.1% had a high school education or below, 16.6% held an associate degree, 17.8% held a bachelor’s degree, and 0.4% held a master’s degree or above. For organizational tenure, 28.2% had less than one year, 27.8% had one to three years, 14.9% had three to five years, and 29.0% had five years or more.

### 3.2. Measurement

All variables were measured using established scales that had been empirically shown to possess strong reliability and validity. Standard translation and back-translation procedures were used to ensure cross-cultural validity. All items used five-point Likert scales (1 = strongly disagree, 5 = strongly agree) except control variables.

Dependence on intelligent machines was assessed through Tang et al.’s (2023b) three-item scale, for example, “I depended on intelligent machines to handle or help with work-related activities.”

Workplace objectification was evaluated using Belmi and Schroeder’s (2021) seven-item scale, exemplified by the statement “I would treat my colleagues as though they are an object.”

Workplace loneliness was gauged through Becker et al.’s (2022) five-item scale, with an illustrative item such as “I feel left out at work.”

Turnover intention was examined via Mai et al.’s (2016) two-item scale, which included statements such as “I will leave the organization within the next 12 months.”

Consistent with prior studies, we controlled for gender, age, education, and organizational tenure to isolate the effects of our core variables. Specifically, gender and age were controlled because these demographic characteristics often influence individuals’ emotional regulation and susceptibility to workplace loneliness(Ong, 2022; S. L. Wright & Silard, 2022). Meanwhile, education and organizational tenure were included as they represent human capital and accumulated organizational commitment, which significantly dictate employees’ alternative job opportunities and subsequent turnover intention (Crossley et al., 2007; Felps et al., 2009).

Cronbach’s α, composite reliability (CR), and average variance extracted (AVE) values were all above the recommended thresholds of 0.7, 0.7, and 0.5, respectively (Fornell & Larcker, 1981; Hair et al., 2019), demonstrating satisfactory internal consistency and convergent validity (Table 1).

## 4. Results

### 4.1. Common Method Bias Examination

Common method bias was assessed using three approaches, considering that the data were derived from single-source self-reports. To begin with, Harman’s one factor test revealed that the first unrotated factor accounted for 43.76 percent of the variance, which is below the 50 percent threshold (Podsakoff et al., 2003). Subsequently, confirmatory factor analyses were conducted to compare models comprising four, three, two, and one factor (Table 2). The four-factor model demonstrated superior fit (χ^2^/*df* < 3, CFI and TLI > 0.95, RMSEA < 0.08), thus indicating that the data met accepted fit thresholds (Hu & Bentler, 1999; Kline, 2015). Finally, a latent method factor model (Podsakoff et al., 2003) showed a slightly improved fit (χ^2^/*df* = 1.953, CFI = 0.968, TLI = 0.961, RMSEA = 0.063). The differences from the four-factor model (ΔCFI = 0.001, ΔRMSEA = 0.001) were below the 0.05 threshold. These results indicate that common-method bias was not a serious concern.

### 4.2. Correlation Analysis

Correlation and descriptive analyses were conducted for each variable. The results are presented in Table 3. Dependence on intelligent machines showed positive correlations with workplace objectification (*r* = 0.203, *p* < 0.01) and workplace loneliness (*r* = 0.239, *p* < 0.001), but its correlation with turnover intention was not significant (*r* = 0.06, ns). Workplace objectification was positively associated with workplace loneliness (*r* = 0.585, *p* < 0.001) and turnover intention (*r* = 0.428, *p* < 0.001). Workplace loneliness was also positively related to turnover intention (*r* = 0.446, *p* < 0.001). These patterns provide a basis for subsequent analyses.

### 4.3. Hypothesis Testing

Hierarchical regression analyses were conducted to test the main effects, controlling for participants’ gender, age, education level, and organizational tenure. As presented in Table 4, employee dependence on intelligent machines positively predicted workplace objectification (*b* = 0.163, *p* < 0.01) and workplace loneliness (*b* = 0.107, *p* < 0.05). Furthermore, workplace objectification was positively associated with both workplace loneliness (*b* = 0.558, *p* < 0.001) and turnover intention (*b* = 0.320, *p* < 0.001). Finally, workplace loneliness positively predicted turnover intention (*b* = 0.358, *p* < 0.001). However, contrary to expectations, the direct effect of dependence on intelligent machine on turnover intention was not statistically significant (*b* = 0.000, ns). Consequently, Hypothesis 1 was not supported.

To evaluate the isolated and serial mediating effects (Hypotheses 2, 3, and 4), we employed Model 6 of the PROCESS macro (Hayes, 2018). In this analysis, dependence on intelligent machine was entered as the independent variable, workplace objectification and workplace loneliness as the first and second mediators sequentially, and turnover intention as the dependent variable, with demographic variables included as covariates. We utilized a bias-corrected bootstrapping approach with 5000 resamples to generate 95% confidence intervals (CI*s*) for the indirect effects. An indirect effect is established as significant if its 95% CI excludes zero.

**Figure 2:**
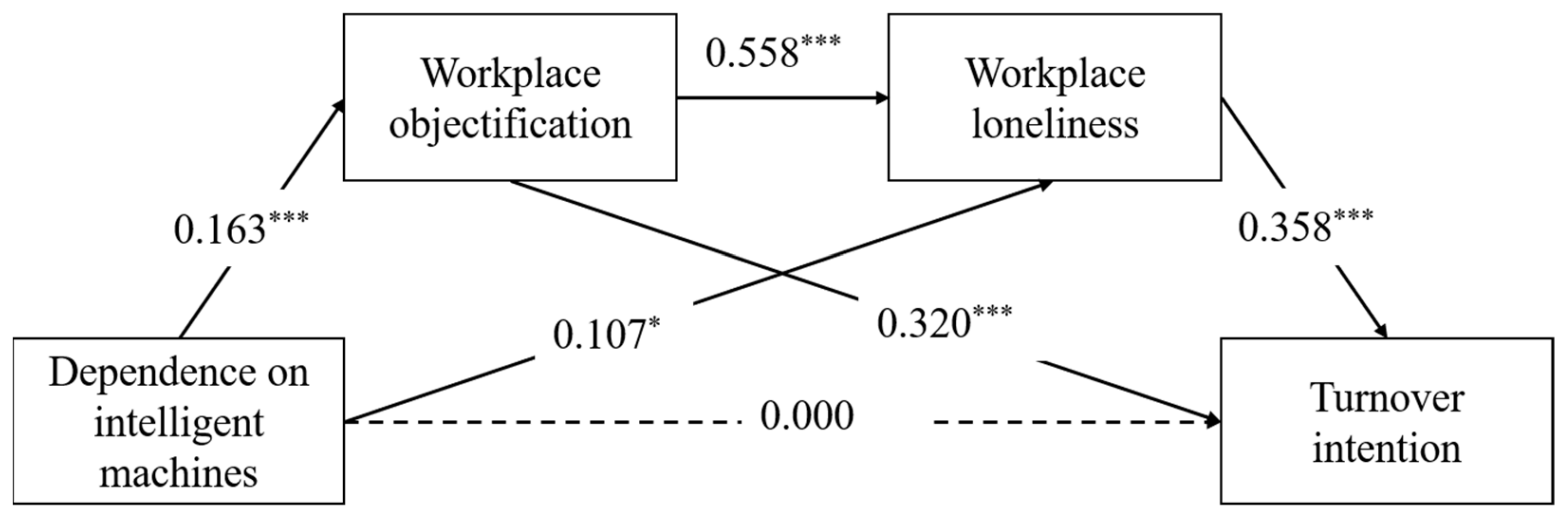
Serial mediation model shows effects on turnover intention. Note: * *p* < 0.05. *** *p* < 0.001 (two-tailed). Solid arrows indicate significant paths, and the dashed arrow indicates a non-significant path.

The bootstrap results (summarized in Table 5) revealed a significant indirect effect of dependence on intelligent machine on turnover intention via workplace objectification (*b* = 0.049, *SE* = 0.022, 95% CI = [0.014, 0.099]), thus supporting Hypothesis 2. Similarly, the specific indirect effect through workplace loneliness was also significant (*b* = 0.036, *SE* = 0.017, 95% CI = [0.005, 0.073]), providing support for Hypothesis 3. Importantly, the analysis confirmed a significant serial indirect effect passing sequentially through workplace objectification and workplace loneliness (*b* = 0.031, *SE* = 0.014, 95% CI = [0.009, 0.061]). This finding supports Hypothesis 4, substantiating the proposed causal chain. The final validated serial mediation model is depicted in Figure 2.

## 5. Discussion and Implications

### 5.1. Main Findings

Grounded primarily in COR theory, this study’s findings clarify how dependence on intelligent machines influences turnover intention through social-cognitive and emotional processes. Results indicate no significant direct effect of dependence on intelligent machines on turnover intention. However, the indirect process from dependence on intelligent machines through workplace objectification and workplace loneliness to turnover intention was significant, confirming serial mediation. The finding reveals a form of path dependency in the effects of dependence on intelligent machines, suggesting that its influence on turnover intention operates through changes in social cognition and emotional experience rather than exerting a direct effect.

The non-significant direct effect of machine dependence on turnover intention offers a lens to decode the efficiency–turnover paradox. As a post-hoc theoretical explanation, intelligent machines provide utilitarian benefits, such as enhanced efficiency and workload reduction, which may offset the psychological costs of diminished autonomy. This proposed offsetting dynamic could effectively mask any direct technological impact on turnover intention. However, because our study did not directly measure these utilitarian gains, this interpretation remains speculative rather than empirically confirmed. Consequently, this offsetting mechanism warrants explicit empirical examination in future research.

However, all indirect effects in our model remain strongly significant. This divergence reveals the true theoretical nature of the threat posed by intelligent machines. The danger does not come directly from the technology itself. Instead, it stems from how technology reshapes the social environment. Dependence on intelligent machines triggers workplace objectification. This subsequent deterioration of interpersonal relationships ultimately drives employees to leave the organization. Therefore, our findings theoretically advance the literature on intelligent machines and employee turnover. We demonstrate that turnover intention induced by intelligent machines is a relational phenomenon rather than a purely technological one. This shifts the theoretical focus from task-level disruption to relational-level degradation.

Aside from the focal hypotheses, educational attainment emerged as a notably strong predictor of turnover intention (Model 5: *b* = 0.521, *p* < 0.001; Model 6: *b* = 0.438, *p* < 0.001). Theoretically, higher education provides employees with greater occupational mobility and more external job alternatives. Furthermore, highly educated workers are particularly vulnerable to perceived over-qualification and over-education, especially in workplaces shaped by intelligent machines (G. Chen et al., 2021; Pan et al., 2025). Such educational mismatches create unfulfilled career expectations that independently increase turnover intention. Importantly, the magnitude of this effect reinforces the robustness of our main model. Highly educated individuals possess a strong baseline propensity for turnover due to market or qualification reasons. Yet, our serial mediation pathway remains highly significant even after accounting for this tendency. This confirms that the relational-level degradation triggered by dependence on intelligent machines acts as a distinct catalyst for turnover intention. It operates independently of employees’ educational background and external marketability.

### 5.2. Theoretical Contributions

This study makes three specific theoretical contributions to the literature on digital work environments.

First, this study clarifies the complex psychological mechanisms underlying the association between dependence on intelligent machines and turnover intention. Rather than proposing a direct technological impact, our findings highlight a specific sequential pathway. The empirical results showed no significant direct relationship between dependence on intelligent machines and turnover intention (H1). Instead, withdrawal behaviors appear contingent upon a chain of social-cognitive and affective resource depletion. By mapping the path from technological exposure to behavioral withdrawal, this study provides a concrete empirical explanation for the paradox of high technological efficiency coexisting with elevated turnover intention.

Second, this research refines the application of workplace objectification within human–machine interaction contexts. Building on previous scholarship that has established the existence of workplace objectification in organizational and AI-related settings (e.g., Andrighetto et al., 2017; S. Chen et al., 2025), this study explores workplace objectification not merely as an isolated outcome, but as a critical cognitive bridge. The findings empirically demonstrate how the instrumental mindset associated with dependence on intelligent machines may spill over into interpersonal domains. This perspective positions workplace objectification as a potential disruptor of reciprocal social exchanges, bridging the gap between human–machine dynamics and deteriorating human–human interactions.

Third, the study enriches the understanding of workplace loneliness in the context of high dependence on intelligent machines. By positioning workplace loneliness as an affective state closely linked to workplace objectification, the research connects cognitive distortions (treating peers as tools) with emotional resource depletion (COR). This nuanced perspective moves beyond simple linear assumptions about technology and well-being. It provides empirical evidence suggesting that turnover intention in digital work environments is fundamentally tied to the breakdown of the workplace socioemotional ecosystem, answering recent calls for clearer examinations of alienation processes in digital work environments (Liu et al., 2025; Susanto et al., 2025).

### 5.3. Implications for Practice

These findings yield several practical implications for organizational management. Initially, organizations must establish a strong supportive foundation by strengthening managers’ techno-humanistic literacy (Osterman, 2019). This literacy helps leaders balance operational efficiency with employee well-being, guarding against an excessive focus on short-term performance. Furthermore, cultivating a culture of human–machine symbiosis ensures that technology consistently serves people rather than subjugating them (Jarrahi, 2018). Building on this cultural foundation, organizations need concrete development initiatives to support employees and disrupt the specific serial mediation paths identified in this study.

To specifically counteract workplace objectification, organizations should integrate thoughtful work redesign (Parker & Grote, 2022) with concrete human resource interventions. Structurally, organizations can delegate standardized tasks to machines. This allows employees to concentrate on creative, emotional, and strategic work. To make this redesign effective at the individual level, performance evaluation systems must also shift their focus. Instead of solely measuring output metrics, evaluations should explicitly recognize and reward uniquely human attributes like emotional intelligence and complex problem-solving. This targeted shift prevents employees from feeling instrumentalized and enhances their perceived work meaning, which reduces turnover intention (Wandycz-Mejias et al., 2025).

To specifically alleviate workplace loneliness driven by high dependence on intelligent machines, organizations must deploy formal social-support mechanisms. Fair communication and inclusive team-building activities are essential baselines. However, organizations should implement more concrete initiatives, such as peer-mentoring programs and cross-functional collaborative workshops, to actively rebuild interpersonal bonds. Additionally, organizations can expand Employee Assistance Programs (EAPs) to offer targeted psychological counseling. Professionals can help employees process feelings of isolation and maintain their workplace identity. By systematically replenishing these social and emotional resources, organizations can effectively mitigate the adverse effects of dependence on intelligent machines on employees.

### 5.4. Research Limitations and Future Research Recommendations

This study has several methodological and contextual limitations that warrant careful consideration and offer directions for future research.

First, the research design limits the ability to draw strict causal inferences. Although a two-wave time-lagged design was employed to reduce common method bias, the temporal separation does not fully align with the proposed serial mediation sequence. Specifically, dependence on intelligent machines and workplace objectification were measured concurrently at Time 1, whereas workplace loneliness and turnover intention were measured simultaneously at Time 2. This structure renders critical links within the serial mediation model essentially cross-sectional, restricting the establishment of temporal precedence. Consequently, the causal ordering implied by our findings must be interpreted cautiously. Future research should utilize three-wave (or longer) longitudinal designs or experimental approaches to rigorously validate the causal mechanisms underlying this process.

Second, constraints regarding sample characteristics and effect sizes may affect the generalizability and practical significance of the findings. The sample size is relatively limited (*N* = 241) and drawn exclusively from the manufacturing sector. Given that human–machine interaction dynamics may vary across industries, future studies should replicate this framework using larger, cross-industry samples to ensure broader external validity. Additionally, regarding sample demographics, age was measured as a broad ordinal variable rather than a continuous one. This categorical operationalization, coupled with a predominantly young sample, may obscure subtle, continuous age-related effects on turnover intention; hence, future studies should collect exact chronological age to capture its variance more precisely. Furthermore, while the structural paths (e.g., from dependence on intelligent machines to workplace objectification and workplace loneliness) are statistically significant, the observed effect sizes are relatively modest. This suggests that while the psychological alienation mechanism exists, organizations should recognize that its practical impact on turnover intention operates alongside other complex organizational factors.

Finally, the reliance on self-report surveys may introduce social desirability bias, particularly for sensitive variables like workplace objectification. Future research would benefit from incorporating multi-source data, such as peer or supervisor ratings. Additionally, the non-significant direct effect (H1) suggests that the relationship between dependence on intelligent machines and turnover intention is highly context-dependent. Future research should examine potential boundary conditions, including employees’ AI literacy, digital leadership, and autonomy-oriented job design. Investigating these factors will help clarify when dependence on intelligent machines is directly associated with turnover intention and when it primarily operates through the serial mediation pathway. Additionally, exploring alternative mediators like perceived skill erosion could yield a more comprehensive understanding of employee withdrawal behaviors in digital workplaces.

## 6. Conclusions

This study explored how dependence on intelligent machines shaped turnover intention through social-cognitive and emotional mechanisms. Drawing primarily on COR theory, a serial mediation model was developed and empirically tested. The results reveal that dependence on intelligent machines has no direct effect on turnover intention. Instead, it influences turnover intention through successive processes of workplace objectification and workplace loneliness.

By integrating technological, socio-cognitive, emotional and behavioral dimensions into a unified framework, this study provides a holistic explanation of how intelligent technologies influence employee behavior. It highlights the paradox of high efficiency and high turnover intention in digital workplaces. The findings suggest that technology shapes employee behavior primarily through a dual process of cognitive distortion and emotional isolation rather than solely through functional mechanisms.

Overall, the study deepens our understanding of how technology-induced work alienation unfolds at the cognitive and emotional levels. It also offers practical guidance for organizations striving to balance human well-being with intelligent-system efficiency. Future research should further refine these mechanisms in a variety of occupational contexts. Such efforts can foster healthier forms of human–machine symbiosis as digital workplaces continue to evolve.

## Figures and Tables

**Table 1:** The results of Cronbach’s α, CR and AVE.

Variables	Cronbach’s α	CR	AVE
Dependence on intelligent machines	0.956	0.956	0.879
Workplace objectification	0.918	0.919	0.620
Workplace loneliness	0.908	0.911	0.676
Turnover intention	0.935	0.936	0.880

**Table 2:** Results of confirmatory factor analysis.

Variables	χ^2^	*df*	χ^2^/*df*	CFI	TLI	RMSEA
Four-factor model (A, B, C, D)	223.565	113	1.978	0.967	0.960	0.064
Three-factor model (A + B, C, D)	981.568	116	8.462	0.741	0.696	0.176
Two-factor model (A + B, C + D)	1292.011	118	10.949	0.648	0.594	0.204
One-factor model (A + B + C + D)	1775.217	119	14.918	0.504	0.433	0.241

Note: A represents dependence on intelligent machines, B represents workplace objectification, C represents workplace loneliness, D represents turnover intention.

**Table 3:** Means, standard deviations, and correlations among variables.

Variable	*M*	*SD*	1	2	3	4	5	6	7	8
1. Gender	/	/	1							
2. Age	1.672	0.772	−0.087	1						
3. Education	1.535	0.796	0.125	−0.310 ***	1					
4. Tenure	2.448	1.183	−0.234 ***	0.508 ***	−0.327 ***	1				
5. Dependence on intelligent machines	3.012	1.096	−0.019	0.154 *	−0.035	0.162 *	1			
6. Workplace objectification	2.483	0.874	0.035	0.012	0.093	0.007	0.203 **	1		
7. Workplace loneliness	2.143	0.878	−0.024	−0.007	0.100	−0.015	0.239 ***	0.585 ***	1	
8. Turnover intention	2.388	1.155	0.062	−0.203 **	0.397 ***	−0.206 ***	0.082	0.428 ***	0.446 ***	1

Note: * *p* < 0.05, ** *p* < 0.01, *** *p* < 0.001 (two-tailed). *N* = 241. *M* and *SD* for gender are not reported as it is a nominal variable. Control variables were measured as categorical/ordinal variables. Gender: 1 = male, 2 = female. Age: 1 = 18–30, 2 = 31–40, 3 = 41–50, 4 = over 50 years. Education: 1 = high school or below, 2 = associate degree, 3 = bachelor’s degree, 4 = master’s degree or above. Tenure: 1 = less than 1 year, 2 = 1–3 years, 3 = 3–5 years, 4 = over 5 years.

**Table 4:** Results of regression analysis.

Variable	Workplace Objectification		Workplace Loneliness		Turnover Intention	
	Model 1	Model 2	Model 3	Model 4	Model 5	Model 6
Constant	2.101 ***	1.716 ***	1.992 ***	0.567 *	1.887 ***	0.500
Gender	0.058	0.052	−0.065	−0.101	−0.004	0.000
Age	0.036	0.012	0.028	−0.008	−0.093	−0.115
Education	0.120	0.112	0.124	0.052	0.521 ***	0.438 ***
Tenure	0.025	0.006	0.000	−0.026	−0.056	−0.064
Dependence on intelligent machines		0.163 **		0.107 *		0.000
Workplace objectification				0.558 ***		0.320 ***
Workplace loneliness						0.358 ***
*R* ^2^	0.012	0.052	0.012	0.363	0.167	0.374
F	0.707	2.579 *	0.713	22.264 ***	11.794 ***	19.882 ***

Note: The coefficient data are unstandardized coefficients. All *R*^2^ values reported are unadjusted *R*^2^. * *p* < 0.05. ** *p* < 0.01. *** *p* < 0.001 (two-tailed).

**Table 5:** Evaluations of serial mediation.

Path	Effect	Boot *SE*	95%CI	
			Boot LLCI	Boot ULCI
Path1: DM → WO → TI	0.049	0.022	0.014	0.099
Path2: DM → WL → TI	0.036	0.017	0.005	0.073
Path3: DM → WO → WL → TI	0.031	0.014	0.009	0.061

Note: DM = Dependence on intelligent machines. WO = Workplace objectification. WL = Workplace loneliness. TI = Turnover intention.

## Data Availability

The data presented in this study are available upon request from the corresponding author.
